# So near, so far, so what is social distancing? A fundamental ontological account of a mobile place brand

**DOI:** 10.1057/s41254-020-00186-z

**Published:** 2020-09-30

**Authors:** George Rossolatos

**Affiliations:** University of Kassel, Kassel, Germany

**Keywords:** Social distancing, Place branding, Experiential consumption, Covid-19, Social phenomenology, Fundamental ontology, Rhetoric

## Abstract

This paper offers a social phenomenological reading of the globally binding practice of ‘social distancing’ in light of the precautionary measures against the spreading of the Covid-19 virus. Amid speculation about the far-reaching effects of temporarily applicable measures and foresights about the advent of an ethos that has been heralded by the media as the ‘new normal’, the ubiquitous phenomenon of social distancing calls for a fundamental ontological elucidation. The purported hermeneutic that is situated in the broader place branding and experiential marketing literatures places Covid-19 in the shoes of Being, and, therefore, imagines how Being would behave ontologically if it were a virus. By arguing that the virus does operate like Being, five theses are put forward as experiential interpretive categories with regard to the ontological status of Covid-19. The adopted approach makes the following contributions to the extant literature: First, it addresses a wholly new phenomenon in place branding, namely a pre-branded place whose meaning is non-negotiable, globally applicable and seemingly equivalent to pure void. Second, it advances the application of phenomenological research in place branding and experiential consumption by highlighting the aptness of the so far peripheral (in the marketing discipline) strand of Heideggerian fundamental ontology. Third, it extends the meaning of place in the place branding literature, by showing how spatialization is the outcome of temporalization, in line with the adopted phenomenological perspective.

## Introduction: why fundamental ontology and why now?

Heideggerian existential phenomenology surfaced at the turn of the 20th C. with the magnum opus *Being & Time* (2001), as a fundamental ontology with an indispensable social orientation (aka a social ontology), intent on subverting both philosophical metaphysics, as well as the cogito-centric approach of Husserlian phenomenology (that was bequeathed in different ways to offshoots/diversions that engage with a cogito-centric platform, including Merleau–Ponty’s corporeal turn (Malpas 2018), Sartre’s existentialism, Schutz and Luckmann’s (1974) social phenomenology). Its interpretive merits, even if someone does not buy the entire intellectual baggage that is offered in Heidegger’s prolific output, consist in pursuing in each instance of the existential analytic a dual orientation. This orientation consists in addressing sociocultural phenomena both in terms of their ontically situated existence, as well as in terms of their fundamental ontological ground, called Being (a quasi-equivalent to god in abstractness, although not the same as regards the concept’s philosophical, rather than theological roots- any criticisms pertaining to the reduction of ontology to ontotheology aside for the time being).

Fundamental ontology is particularly pertinent in the face of increasingly uniform globalizing discourses that seek to bring about a univocal, all-binding ethos (what is trumpeted as the ‘new normal’) in the time of Covid-19. This is a temporal moment in the history of mankind that suspends all activities as we know them in the face of a menacing force that is manifested as a lethal virus. The magnitude of the sweeping effects of this virus legitimates us in claiming that in the time of Covid-19 Being has been replaced with a virus. In this temporal suspension from becoming as we knew it, new cultural practices have been introduced, while current ones are constantly being reframed.

The newly introduced practice of social distancing which amounts to the institution of a ‘dead zone’ (as will be called later on), as spatial distance between subjects in public space, not only afforded to redefine how we interact in public, but also what is entailed in transgressing the boundaries of this mobile space.

Social distancing is particularly pertinent for place branding, as it opens up a wholly new way of experiencing intersubjectively public space. This novel experiential mode is further substantiated by the following: (i) the ubiquity of this spatialization strategy (ii) the mobility of this space in-between subjects (iii) the utter absence of any symbolism that renders it akin to an experiential void, i.e. it is diametrically opposed to, for example, memorial spaces that are responsible for construing memories based on overt symbolism; there is ‘nothing to be seen’ in this space (iv) its univocal branding as a no-go zone that potentially harbors the virus whose meaning is not negotiable by individual brand associations (v) the institution of a new topology with bespoke geographical boundaries that transcend received demarcations, i.e. the virus has an autonomous modus operandi and its own spatialization rationale.

The cultural practice of social distancing or maintaining one’s distance from another in physical space, constitutes an inviolable mandate whose meaning overshadows that of a mere social norm. Social distancing is a matter of life or death. Therefore, social distancing is by default not just a functional issue in ordinary social interaction, or an ontic issue (in Heidegger’s terms), but a social ontological issue that defines in a fundamental manner being-with in public space. Theorizing the implications of this mandatory spacing on an ontological level is, therefore, of primordial importance in understanding how essential aspects of socialization are being redefined by Covid-19 as the now/new Being.

In light of the above introductory remarks, this paper pursues the following argumentative pathway: An overview of key concepts of Heidegger’s fundamental ontology is offered as the conceptual scaffolding for the ensuing existential phenomenological analytic. This is followed by a discussion that is intent on situating fundamental ontology among the prevalent perspectives in contemporary place branding theory and in justifying its relative merits in understanding Covid-19′s spatialization strategy and the meaning of social distancing as lived experience. Then, five theses are presented as regards the macro-cultural implications and the social functions of the in-between space that marks the boundaries of social interaction in the time of the pandemic. Finally, the analysis wraps up by pointing out the implications and further applications of this study in a broader technoscientific regime.

## Key concepts in Heidegger’s fundamental ontology

Claiming to be offering even an introductory overview of Heidegger’s voluminous conceptual armory in a single article section is bound to loom like a lethal deathrow with a river monster. This section performs a more humble task by setting out to familiarize marketing researchers who are not necessarily versed in Heideggerian ontology with a limited set of concepts, such as the ontological difference between Being and beings, being as existence (Dasein), space and time, being-towards-death, authenticity/inauthenticity, ready-to-hand/present-at-hand. These terms are narrowly relevant to appreciating how this perspective attains to shed light to the meaning of the lived experience of void space in the practice of social distancing. Three seminal works by Heidegger are mainly drawn upon that are directly relevant to space and time, namely *Being & Time* (2001)*, Time & Being* (1972), and *The basic problems of phenomenology* (1988). Also, it should be stressed at the very beginning of this conceptual exposition that Heideggerian ontology is not endorsed here in an uncritical manner. This will be demonstrated both here and in the final section that questions fundamental tenets from critical theoretic and deconstructive angles, aimed at understanding key aspects of the philosophical perspective’s rhetorical structuration.

With these provisos in mind, it must be established at the very outset that Heidegger’s ontology is highly anthropocentric. At its kernel lies the human being that is defined as existence or *Dasein* (in German), literally meaning being-there (‘sein’ and ‘da’). This ‘thereness’ constitutes the place of truth for human subjects, a truth that always withstands appropriation by a subject as full presencing. Spatiality, thus, is of central importance in understanding the essence of human beings. This essence (roughly speaking) rests with Being or what appears each time that we ask ‘what is X.Y.Z?’ Being is presupposed in every sentence uttered by beings. Therefore, any investigation (e.g. cultural, philosophical) about what it means to be human is conditioned by Being that presents itself in the copula ‘is’.

The space of ‘thereness’ is metaphorical, or, in Derrida’s terms, archi-metaphorical as it buttresses the entire phenomenological edifice of fundamental ontology. The difference between Being and beings was termed by Heidegger the ontological difference. This does not amount to the difference between a transcendental god and finite humans, but, in a highly anthropocentric manner, to the horizon of possibilities that ‘is’ Dasein. In other words, Being is human existence *as* potentiality-for-Being. It denotes an open horizon for fulfilling one’s potential (and not a super-human entity that is capable of monitoring ordinary affairs, while refraining from intervening in them).

Ontological difference also displays a spatialization rationale, insofar as it points to a distance between Being and beings. This distance is experienced as a constant nearing and distancing, insofar as Being is at the same time nearby beings as totality and horizon, and far from beings, as realized potentiality-for-Being. This impossible approximation institutes a paradox at the heart of existence, namely that for as long as a subject is, it may not reach Being, however without being enmeshed in the process of approximation it may not be as such. Dasein is always already caught up in this paradoxical space. Space, here, is not intended as a metric system for a tactile container and as mere *res extensa*, but as existential space, always in a process of spatialization. This is a very important aspect of fundamental ontology, of direct pertinence to place branding, as will be further illustrated in the ensuing section. The relationship of beings to their Being is experienced as spatialization in terms of constant nearing and distancing. In other words, what is near is always already far from being approximated as such.

The underlying distinction in the preceding sentence concerns another fundamental aspect of Heideggerian ontology, namely that between authentic and inauthentic existence. Existing authentically involves heeding Being in an unadulterated manner, that is as pure form, whereas existing inauthentically involves being oblivious to the question of Being, while being enmeshed in ordinary affairs, or, as put by Heidegger (2001), lost in the Mit-sein (among the anonymous ‘they’ in ordinary idle speech). Although Heidegger claims that this distinction is not intended to devalue practices such as reading fashion magazines, for example, it does manifest an underlying tendency at valorizing speculative philosophical discourse over empirical discourses. This speculative distinction is axiomatically posited as such, and has been vehemently criticized by critical theorists, such as Adorno (1973), as the jargon of authenticity (also notice that Krell (2015) opts for using the terms ‘appropriate’ and ‘inappropriate’ instead of ‘authentic’ and ‘inauthentic’ which is a more accurate translation in terms of conveying that the ontological question lies closer to the ‘proprius’ of Dasein compared to ontically grounded ones; also see Inwood 1999).

From a critical standpoint, it may be counterargued that Heidegger’s latent valorization of vacuous formalism amounts to living in a constant state of negation, or in adopting a negatively nihilistic posture (in Nietzche’s will-to-power terms). Heidegger, of course, claims the opposite, namely that being enmeshed in ordinary affairs amounts to a forgetfulness of Being, to residing in a state of oblivion, even going to such extremes as to almost vindicate humanity for being ‘thrown’ into everydayness (apparently from some sort of utopian paradisic [parasitic] being fully present to oneself). From a relativist point of view, either side in this dialogue is legitimate, and ultimately boils down to whether one prefers to live as a monk or as a politician, without precluding that monks may also be politicians, as attested by figures such as Dalai Lama, the Pope, the Kung Fu master (starring David Carradine).

In any case, living inauthentically is an inevitable condition of human existence, according to Heidegger, and it is through fissures in a cultural inauthentic fabric that beings are summoned to respond to the overarching question of Being. Such fissures include instances where practical equipment breaks down. In these instances, what is practically proximate as ready-to-hand exposes its instrumental rationale (Rae 2010) as equipmentality, and at the same time its facticity as part of a praxiological nexus. It is only when a tool becomes divested of its functionality that it emerges as present-at-hand. In our case, a car’s readiness-to-hand as transportation means broke down in the face of a globally enforced lockdown, during which civilians’ out-of-home movements were prohibited. In this predicament, cars were divested of their habitual meaning as parts of a cultural nexus, and distinctive sign-systems, while being transformed to present-at-hand objects. And what this presencing shows is the unconditional (in instrumental terms) co-belongingness of beings with other beings and with objects in an ontological milieu. “Both subject and object are thereby ‘placed’ within the same structure, rather than one or the other being the underlying ground for that structure” (Malpas 2018, p. 35).

This distinction between authenticity and inauthenticity is carried over to temporality which, in fact, is more primordially related to being’s Being compared to spatiality. This may be demonstrated with reference to the process of spatialization, as above described. In order for space to be spatialized, i.e. to be embedded in a universally meaningful formation, it requires time. Time as horizon of temporality conditions Dasein’s existence fundamentally, as well as being-in-space. “Heidegger’s prioritization of temporality in *Being and Time* takes the form of an assertion of temporality as the originary *foundation* for the unity of *Dasein* in its entirety” (Burdett 2015, p. 115). “The ontological condition of the possibility of the understanding of being is temporality itself” (Heidegger 1988, p. 228).

To make this argument more palatable, think of movement in pure void, without any tactile ground, as in outer space. Still, movement in outer space requires time, despite the fact that no ground, strictly speaking, has been covered. The real reason, though, behind Heidegger’s prioritization of time over space is that since the existence of Dasein is tantamount to potentiality-for-Being, beings *are* always ahead of themselves as yet-to-come or as unrealized potential. This yet-to-come presupposes a future tense, as well as the possibility of this future to be envisioned from a present and a past, as “existential projection or fore-casting of Dasein as possibility-being” (Krell 1986, p. 32). This is why not only temporality is the foundation of space and the condition for spatialization, but authentic temporality is equivalent to the co-presence of past, present and future. Each temporal mode constitutes, then, a temporal ec-stasis, as termed by Heidegger, literally meaning (in Greek) standing (‘-stasis) outside (‘-ec’) of oneself. And since Da-sein is always being-there, temporality as the horizon of becoming of beings as beings-there is the primordial phenomenological way of existence. This will be further illustrated with reference to the time of Covid-19 in the section after the next one. Although Heidegger prioritizes the future among the three temporal ecstases in his early work *Being & Time* (2001), in his later work *Time & Being* (1972) presence is posited as the most eminent mode.

The final conceptual component that is essential in this hermeneutic endeavor concerns Dasein’s existence as fundamentally being-towards-death. This is intimately interwoven with Dasein’s future orientation. Death, here, is used as a synonym for ‘end’ or ‘teleology’, as conceived by Aristotle, that is ‘telos’ as end-point, and at the same time as objective conditioning teleologically the process of becoming (see Rossolatos 2018a,b). The paradox that lies in the heart of spatialization as contemporaneously nearing/distancing, and of existence as triply ecstatic, also applies in this instance. The answer to the question of Being will have been furnished at the point where being will no longer be capable of articulating it (i.e. the moment of physical death), whereas for as long as beings are their death as teleological horizon is in question.

In conclusion to this section, a short commentary on the rhetorical construal of fundamental ontology is offered, for the sake of both pre-empting perceiving Heidegger’s perspective as a canonical social ontological blueprint, and facilitating the transition that will be made later from Heidegger’s essentializing discourse towards a rhetorical topology that views space, time, and social distancing as metaphorical constructs or literalized metaphors (Mitchell 2012, 2017). As pointed out by Gross (2005), Heidegger relocates rhetoric at the heart of his fundamental ontology.

In this respect, the ontological difference between Being and beings amounts to a sublimation of the verb ‘to be’ that is displaced from its ordinary grammatical contours and posited as ideational existential depth. Heidegger’s recurrent rhetorical ploy follows three moments: initially, the aboutness of an assertoric statement that employs the verb ‘to be’ is problematized by making the assumption that in order to understand what is referred to, we must first obtain an understanding of the verb ‘to be’. Then, the question of what is that which precedes any ontical determination (as grammatical phenomenon) is catapulted to an overarching existential question. The grammatical origin of the speculation recedes in the background and from that point onwards the human condition is identified with a phenomenology of Being as autonomous ‘entity’ (yet intimately related with beings).

This autonomization of Being is evinced most eminently in the employment of the middle voice in the description of the dialectic of truth. The dialectic of truth is not a process that is carried out by humans based on the exercise of free will. Humans do not disclose truth which would force the aletheic process to lean on the agentic side as truth-finding. Rather, the process of disclosure is presented with the employment of middle voice as a ‘bringing forth from concealment’, that is as a self-subsistent process that is neither fully motivated by beings, nor imposed onto beings by some sort of transcendental entity, as Being (also see Heidegger 1972, p. 8; Desilet 1991 on the translation of ‘es gibt’ as ‘it gives’). In fact, as Derrida contends, it is questionable whether Being is anything else than an “ontic metaphor struggling in vain to become ontological conceptuality” (Krell 2015, p. 90). As also pointed out by Karrer (2006, pp. 5–7), “Heidegger's basic metaphors are territorial (ground, horizon, region, district) […] Border rhetoric uses limit, border, and boundary interchangeably, and mixes it with words of closure/openness, experience and other existential or interstitial words to achieve a mystifying theoretical-sounding effect which these words carry over from their semantic history.”

Upon sketching out the key concepts from Heidegger’s fundamental ontology that will be recruited in the offered account of social distancing, the following section demonstrates how this perspective complements and extends the ontology of place branding in light of the scrutinized phenomenon.

## Situating fundamental ontology in the contemporary place branding landscape

Although the place branding literature is conceptually rich from a modeling point of view, considerably less reflection has been allotted to ontological considerations of space and place. In this section, the Heideggerian ontological perspective on spatialization is situated amid the dominant perspectives in place branding, and its relative merits are discussed.

Each of the aforementioned strands shares different ontological assumptions about place brands. In particular, the identity perspective views places as assemblages of promotional discourses and identity claims. The associationist approach reduces the meaning of place to the nexus of brand associations in the minds of various stakeholders. The narrative approach identifies places with the stories and texts (Hanna and Rowley 2008; Rossolatos 2020a, b) that circulate about them. The interactionist approach identifies places with the collective constructions of the meaning of places that shape up through social interactions. Lucarelli and Giovanardi (2016) added a fifth perspective, the mobilities one (although it may be viewed tentatively and tenuously as an extension of the interactionist approach). The mobilities perspective emphasizes the construal of space through bodily movement. “The new mobilities paradigm represents an approach that is underpinned by a relational ontology as it promotes an understanding of society and the lived experiences of people (and consumers) not as fixed and given but as constantly played out from the relationships between their movements and trajectories” (Lucarelli and Giovanardi 2016, p. 324). Finally, Kavaratzis and Ashworth (2015) suggest a culturally inflected meaning of place as a set of elements which may be picked by consumers to mold a place culture as they choose. The enculturation process has also been posited by Florek and Kavaratzis (2014) as central to the transformation of meaningless, physical space into meaningful place. In this respect, places have been identified with social constructs and assemblages (Warnaby and Medway 2013). Most importantly, Warnaby and Medway (2013, pp. 348–349) suggest three ways whereby place has been contextualized: location, locale and sense of place. The latter incorporates the temporal dimension, that is the primary dimension wherein cultural practices are enacted according to Heidegger, albeit in an inauthentic manner, and, hence, ontical, not ontological.

The above identified perspectives, with the exception of the mobilities and the text oriented ones, assume latently a subject/object divide, even when not explicitly admitting it. The mobilities perspective reverses the priority between body and mind by assigning agentic status to corporeality, thus lying closer to a Merleau–Pontyan perspective. Heidegger would be quite aversive to such a reversal, especially given that it amounts to retaining the Cartesian distinction between res cogitans and res extensa on the inverse which he overcame with the paradigmatic shift from subject to Dasein.

Lucarelli and Borbstrom (2013) attempted a synthesis of the place branding literature by drawing on the ontological/epistemological criteria that were used by Burrell and Morgan in their model of sociological paradigms. According to this model,

studies adopting an objectivist approach, whether they are radical structuralist or functionalist, share an ontological (realist) and epistemological (positivist and post-positivist) standpoint, usually adopting a nomothetic methodology and a determinist point of view about human nature. Studies adopting a subjectivist approach, whether they are radical humanist or interpretivist, are ontologically nominalist and epistemologically relativist (social constructionist) (Lucarelli and Borbstrom 2013, p. 71).

Subsequently, Lucarelli and Borbstrom (2013) perform their synthesis based on the axis of objectivism/subjectivism which culminates into six types of place branding research, namely the critical structuralist, the radical humanist, the production, the co-production, the consumer-oriented and the appropriation perspectives. This is an interesting endeavor and beyond any doubt a scholarly undertaking that paves the way for critically engaging with epistemological and ontological issues that more often than not remain unaddressed in place marketing and branding conceptual and empirical research. However, it is not clear why the authors, on the one hand, selected Burrell and Morgan’s framework which tends to aggregate quite heterogeneous perspectives under individual types, and, on the other hand, how they categorized the selected place branding perspectives under the objectivist/subjectivist pair (especially given that the categorization is presented in the final section of the paper without any allusion to specific features from the concerned papers). This categorization would require a more explicit display of the underlying conceptual components, given that in the majority of instances the mentioned authors do not assume explicitly epistemological and ontological positions. But, most importantly, this classification perpetuates the Cartesian distinction between res cogitans and res extensa, while resting on the assumption that objectivist accounts of place branding amount to a correspondence between how place is lived and how it is in itself, i.e. in its materiality as res extensa.

The only classification, to my knowledge, of place construals that lies closer to the Heideggerian project than the rest has been put forward by Cresswell (cited in Warnaby and Medway 2013, p. 349). This typology features three approaches to the meaning of place, namely descriptive, social constructionist, and phenomenological.

The descriptive (or ideographic) approach postulates that the world is a set of places that can be studied as unique and particular entities. The social constructionist approach views place as the progeny of underlying social processes. The phenomenological approach seeks to define the essence of human existence as being necessarily in “place”. The meaning of being in, phenomenologically speaking, was analyzed in the previous section from a temporal point of view.

On a very different note, the main contributions of Heideggerian fundamental ontology amid the multivocal conceptualizations of space and place branding consist in the following: (i) space is dislodged from the subjectivism/objectivism binarism which is premised on an object ontology (i) for the first time space is viewed dynamically as existential spatialization (ii) this process is intimately interwoven with temporalization, as the condition of spatialization (iii) space is explicitly relativized in terms of proximity by assigning primacy to a ‘thereness’ as the place where Dasein is bound to be, yet which for as long as it is cannot be presenced in fullness.

In an attempt to anchor more succinctly Heidegger’s take on spatialization in the broader marketing literature, with the aim of overcoming ossified binarisms such as the objectivism/subjectivism divide in place branding, the expository focus will now turn to the experiential marketing literature.

## Points of convergence between experiential marketing research and Heideggerian phenomenology

Phenomenology in general and phenomenological strands in particular have received scant and piecemeal attention in the broader consumer research and branding literatures. Nevertheless, their value in reorienting attention from individual psychological processing mechanisms to more immersive aspects of consumption has been noted time and again (e.g. in the case of entertainment marketing by Hackley and Tiwsakul 2006). It is in such a contrastive mode with the entrenched marketing research vernacular that focus on the experiential aspects of consumption was laid ever since Holbrook and Hirschman’s (1982) seminal publication on consumer fantasies, feelings and fun (also see Holbrook 2018).

Interest in phenomenology has been growing ever since (Thompson et al. 1989), albeit to a greater extent as qualitative research method (i.e. the phenomenological interview, cf. Becker 2018), in line with its increasing importance in the methodological roster of qualitative inquiry across disciplines and the popularization of techniques such as imaginative variation and the phenomenological (transcendental) reduction, rather than as distinctive strands, equipped with a rich conceptual armory for performing cultural hermeneutic readings (Hirschman 1986; Thompson et al. 1994; Elliott 1997), especially of extraordinary consumptive and branding phenomena. And even in this limited context, Husserlian phenomenology has tended to be the norm, primarily due to its being neatly aligned with contemporary cognitivistic and psychologist perspectives- despite the fact that overcoming psychologism and rooting the social sciences in *philosophia prima* was Husserl’s expressed objective in coining transcendental phenomenology. On the contrary, Heideggerian phenomenology has been lying at the very periphery of research interests (cf. Horrigan-Kelly et al. 2016), not in the least due to its considerable conceptual complexity that occasionally comes across as utter obscurantism to the non-philosophically inclined.

Nevertheless, Heideggerian phenomenology as conceptual edifice and methodological toolbox for analyzing the ramifications of fundamental ontology is tailored to the exigency for overcoming the time-hallowed subject/object binarism that largely informs marketing research (Thompson et al. 1994). This binarism inheres in the broader marketing ontological terrain. As noted by Grassl (1999, p. 3), “idealists about brands see perceptual or cognitive acts of consumers grouped under the heading ‘brand awareness’ or ‘brand image’ as constitutive for the existence of brands […] Realists, on the other hand, reject the view of brands as mere marks or names and inter-pret them as emergent products with properties that afford branding.” Overcoming the binarism is attainable by adopting a praxiological (Schatzki 2000), rather than an epistemological approach, in line with the existential analytic that deploys in *Being & Time* (Heidegger 2001).

The existential analytic aims at “bringing to light the basic structures of Dasein” (Heidegger 1988, p. 227), prioritizes meaning over epistemological criteria (and its coupling, that is object ontology) and situates both subjects and objects in a vast nexus of cultural practices wherein they are inauthentically enmeshed. This does not imply that objects have the same ontological status as subjects, as the much popularized assemblage theory might contend. On the contrary, according to Heidegger’s explicit anthropocentric stance, humans are uniquely equipped both compared to other animals and objects for attending to the meaning of Being, mainly due to their being endowed with the gift of language. As shown earlier, the essence of Dasein’s existence is not exhausted in its relationship with ready-to-hand objects, but rests fundamentally in an horizon of possibilities which is transcendental with regard to their situated enmeshment in ordinary affairs. The essence of Dasein stems from this web of meaning that is presided (so to speak) by Being. It is the totality of this meaningful whole that is impenetrable to humans.

Experiential marketing converges with the praxiological orientation (Gahrn Andersen 2019) of Heidegger’s existential analytic insofar as it seeks to explain consumption by drawing not on epistemological categories and object ontology as their correlate, but by coining wholly new categories that are sensitive to immersion (rather than distanced understanding). As noted by von Eckartsberg (1998a), in experiential marketing “the emphasis is on the study of lived experience, on how we read, enact, and understand our life-involvements.” The same orientation is shared by the application of phenomenological research tools in qualitative inquiry (Cresswell and Poth 2018).

Most importantly, in experiential marketing “interpretive scholars have illuminated further the nature of extraordinary experiences (e.g., intense leisure activities)—referred to, as we saw, as “peak experiences” (Privette 1983), “epiphanies” (Denzin 1992), or transcendent customer experiences (Schouten, McAlexander and Koenig 2007). They found that extraordinary experiences are achieved through intense and focused activity, and absorption or immersion in those activities” (Schmitt and Zarantonello 2013, p. 45). Ec-stasis, as noted earlier, is a fundamental trait of Dasein’s ontological constituency. Dasein does not have to engage in a specific type of experiential activity to affirm its essence as always being outside of itself in a dialectical relationship with the disclosure of its thereness. This is a fundamental ontological condition as constant nearing and distantiation. In this sense, stressing that ‘transcendent customer experiences’ (TCE) constitute a specific type of consumption, “including feelings such as self-transformation, separation from the ordinary and mundane, and connectedness to larger phenomena outside one’s self” (Schouten et al. 2007, cited in Schmitt and Zarantonello 2013, p. 46) is ontologically superfluous.

The same holds for relational experiences, defined in an experiential marketing setting, albeit whose definitional contours are not phenomenologically informed, as “emerging from social contexts and relationships that occur during common consumption as part of a real or imagined community or to affirm social identity” (Gentile et al. 2007, cited in Schmitt & Zarantonello 2013, p. 43). Dasein is ontologically relational by definition (de jure), and not contingently so (de facto).

On an even more concrete level, fundamental ontology meets experiential marketing, in a paradoxically divergent encounter, in the way near-death experiences are theorized by the latter. “Even death is seen as part of life. As one of the skydivers interviewed put it, ‘we do not have a death wish, we have a life wish!’” (Schmitt and Zarantonello 2013, p.46). In this instance, being-towards-death is employed as an experiential category by experiential marketing, albeit in an ontic, empirical manner, rather than as a fundamental ontological structure (as shown earlier).

Fundamental ontology, by dint of addressing each phenomenon on both ontic and ontological levels is capable of accounting for the macro-cultural meaning of cultural practices such as social distancing, by making visible what may not be seen in plain view (e.g. what inheres in an empty space in-between subjects). In this manner, the distinction between authenticity and inauthenticity concerns how ontological structures may be unearthed through mundane experiences (what Adorno (1973) called- albeit critically- the *fundamentum in re*), and not a hierarchical stratification from less to more authentic where inauthentic denotes “the sense of meaninglessness and superficiality in modern society” (Firat and Venkatesh, 1995, cited in Schmitt and Zarantonello 2013, p. 33). This intuitive employment of inauthenticity obfuscates the principle that what is authentic and what is not, lies in the eyes of the beholder, i.e. it is a contingent, radically empirical judgment, without any trace of necessity (even more so of ontological gravity).

What is noteworthy, though, is the privileging in experiential marketing of categories that are capable of capturing consumptive experiences as such, rather than reducing them to brand associations stemming from experiencing, thus propagating the distinction between res cogitans and res extensa (the territory of experiential benefits, according to Keller, and of experiential value, according to Schmitt and Zarantonello 2013). It is in this mindset that the lived experience of the in-between space in the enactment of social distancing will be encapsulated in five experiential interpretive categories in the following section.

## Five theses on social distancing from a fundamental ontological point of view

### The nearness paradox-social distancing harbors an ontological paradox: what lies nearby Dasein ‘is’ at the farthest extremes of its possibilities

“The shortening of distance, nearness, and remoteness condition our existential and thinking modalities” (Koupannou 2018, p.105). Social distancing implies the prohibition of physical proximity between at least two Daseins in public space. However, this inauthentically mandated distancing, i.e. a distance that abides by a metric system whereby natural space may be measured, also implies the Daseins’ physical proximity to an empty space that lies in-between. This at once distancing and the removal of distance has been called by Heidegger de-severance (cf. Eiland 1984). De-severance opens up a mobile space that is construed in interaction and as Daseins move in space, and assumes meaning seemingly due to being meaningless. It is just a void that separates two Daseins or a piece of land that is ready-to-hand in light of the cultural practice of social distancing. As per the initial exposition of the distinction between ready-to-hand and present-at-hand, it is precisely the breakdown of the ordinary meaning of an object that allows it to be theorized authentically, from a fundamental ontological point of view. For example, a spatial territory outside of a supermarket that surrounds the entrance, is customarily used as the pathway to the store. This pathway is now interrupted by multiple fissures that have disrupted the customary signification of this pathway. This emptying is tantamount to what Heidegger called ‘clearing’, that is an abstract space that is central to the constitution of the social (Schatzki 2000). In this abstract space lies being-towards-death as potentially being lethally contaminated by the virus. Insofar as death is equivalent to Dasein’s Being (Adorno 1973, p. 139), maintaining intersubjective distance is tantamount to being near one’s thereness. “Disclosedness, and not the shortness of physically measurable distance, is the true meaning of nearness” (Chaudhary 2020, p. 10). This proximity propagates the relationship between Dasein and death, insofar as to actualize one’s potential, the limits of the interim space must be crossed. Since such a crossing would be suicidal, what is most proximate to Dasein must be kept at a distance, a paradox in the heart of social distancing.

### The deadzone-the interim mobile space is a deadzone that harbors death as Dasein’s ownmost potentiality-for-Being

Social distancing essentially boils down to a reification (i.e. realization of a theoretical construct) of the ontological difference between Being and beings by demarcating a deadzone in social interaction as essential condition of being-with. This deadzone fleshes out simulacrally authentic temporality in a mobile and omnipresent hic et nunc. Therefore, what presences itself in the interim space near which Daseins are proximally placed in public space is Being as no-go zone and taboo space. This omnipresent interim space condenses ecstatically past and future. It is a post-apocalyptic simulacrum that inscribes death in-between subjects in a new cultural order brought about by the time of Covid-19 that revolves around a fundamental thanatography. The deadzone is an omnipresent place that moves alongside beings, in the same fashion that Being, according to Heidegger, appears alongside beings. This mobile space is construed in co-ordinated movement of at least two subjects in public space. This is not an ordinary sign-post (e.g. caution: wet floor), but the ultimate sign-post: this is the taboo space of death. The mobile spatialization of this taboo space is equivalent to the inscription of death in social space as fundamental condition for being-with ontologically. Moreover, the meaning of this place is not dependent on individual brand associations, as per the associationist perspective on brand equity in general and place branding in particular, but is univocally and universally associated with death as possibility. Therefore, it is a fundamental social ontological condition.

### Being-with nihilistically-the interim mobile space is an existential copula that unites subjects in their distancing via a void that stands for nihili locus

For Heidegger, existence is essentially intersubjective, as being-with (McMullin 2012). It is through the we-relation that the intersubjectivity of the lifeworld is developed and confirmed (Schutz and Luckmann 1974). Social distancing as mutually presupposing distance from one’s Being as horizon of possibilities procurs a set of co-ordinates for being-with in public space. On an ontical level, this co-ordination concerns the pragmatic need for regulating intersubjective movement and is incumbent on maintaining distances between each other as mutual proximity to an interim space. This in-between space marks ontologically, i.e. unconditionally with regard to ontic determinants (any space becomes retrajectorized according to the demands of the virus) being-with in proximity. The reciprocity of beings originates out of the between (Theunissen 1984). At the same time, this space harbors subjects’ potential annihilation. Therefore, the in-between mobile space that spatializes the practice of social distancing and is spatialized because of it, as a “practice of relationality and connectivity” (Lury et al. 2012, p. 12), is premised on annihilation due to the omnipresence of the virus. Being–with as being distanced in social space amounts to being proximate to an interim space as nihili locus.

### The spectral space-the metaphysics of presence has been substituted by a metaphysics of absence in a ghost space

Being is sustained in its materiality in the interim space that spatializes social distancing as the virus that is as imperceptible (in plain view and sensing) as Being (as intangible construct). Yet, it performs the same social function as the fissure in the social text that unites social actors precisely as impossibility of full presencing. In this manner, the interim space that conditions social distancing constitutes a metaphorical space, spatialized by an epidemiological trope (Mitchell 2017). This metaphorical space has been branded according to a rationale of spectral play (Vanolo 2018). What is housed in this spectral space is not absently present, but presently absent, i.e. a nanoparticle that stands for death as the end of Dasein’s potentiality-for-Being. The virus does not have to actually be in the deadzone to perform its thanatohraphic role. If it is actually there, it cannot be seen in plain view, and if it is not there as a natural entity, it is still there in virtual state by dint of the spatialization strategy that shelters it. This is what Derrida called fantasmatic ontopology, as an ontological ground in topology (Phillips 2013; Burdett 2015) that is not static, but whose form is constituted in movement (Lury et al. 2012). “It is useful not to conceive the visible and the invisible as two distinct and separate spheres. By mobilizing the idea of the ghost, it is possible to think of a further plain situated in an intermediate position between the visible and the invisible” (Vanolo 2018, p. 56).

### The subversion of being by technology-the interim mobile space is a rhetorical space that harbors a crime scene: the death of Being as the casualty of a lethal nanoparticle

As may be gauged by recourse to Heidegger’s prophetic effacement of the notion of distance in *Time & Being* (1972) with the proliferation of modern technologies (online discussion and teleconferencing platforms being a remarkable case in hand), the future is already here. If technology performs an integral role, for Heidegger, in projecting Dasein’s possibilities to the farthest corners of its horizon, and if this projection is an indispensable part of Dasein’s existence, then we have already stepped into a post-Being era where such infinite possibilities are, if not yet materialized, at least feasibly foreseeable. The central thesis that is propounded at this juncture is that Covid-19 constitutes the inauguration of this post-Being era, and that social distancing constitutes a mode-of-being in the world alongside others that is overdetermined by a moving interim space where this already appropriated post-Dasein future is engrafted.

The post-Dasein ascription pertains to the man-made (lab-made, more aptly) nature of the virus, yet an artifact that is not of the same order as ready-to-hand objects that abide by the instrumental rationality of equipment, as described by Heidegger (2001). It is an artifact that stands metonymically for Death, as the exhaustion of Dasein’s horizon of possibilities or the end of the horizon. In the face of this artifact, the entire spectrum of Dasein’s ontic structures have been radically redefined which attests to the artifact’s equiprobable status as that of Being’s. At the same time, this artifact (as yet) is as inscrutable as Being.

To recapitulate, if technology, as envisaged by Heidegger, has the potential to radically redefine Dasein’s relationship to its horizon, thus taking the place of Being, then, at this juncture this replacement has already taken place. This is not an epochal replacement, as approximation and distancing of Dasein from its historical deployment in a uniform horizon that is delimited by Being, but a rupture with Being and the dawning of a new era where Being has been replaced by a technoscientific regime (Ihde 2009). In the same manner that being-towards-death is Dasein’s ownmost potentiality-for-Being, being in proximity to Covid-19 as distance from another Dasein delimits an ontological territory where technology, in all its devastating potential, is inscribed as the new limit.

## Conclusions

Arguably, social distancing constitutes a reification of Heidegger’s speculative rhetoric, a typical instance of fleshing out don quixotically a philosophical fabula. This may be shown most eminently by attending to how the ontological dimension becomes a necessary ground for ontic existence, that is through the tropicalization of the process of Being’s presencing. This process is grammatically construed with the employment of the middle voice which suspends the ascription of agentic status behind this presencing either to beings, as inauthentically enmeshed social actors, or to Being, as potentially being conflated ontotheologically with a transcendental entity (in a religious sense). This rhetorical stratagem was employed in a terrain where language and verbal communication were preponderant over other modes. Therefore, the quest for primordial meanings behind inauthentic practices, including linguistic ones, was undertaken by Heidegger by tracing genealogically and reviving archaic ways of lexical use, re-purposed in the context of the system of fundamental ontology. Nevertheless, Heidegger’s constant preoccupation with hyphenation in an attempt to capture the ‘world’s worlding’ in flux is indubitably exemplary and conducive to the furtherance of neologistic plasticity, for example by coining the term being-telepresent-at-hand.

In a technoscientific regime, situated in a post-ideological milieu where other modes, such as proxemics and kinetics, have also taken over the linguistic, the necessity of the ontological emerges in the context of a relational, mobile spatialization that leverages humans as participants in a ubiquitous micronarrative of life and death. What is presenced in the interim space that conditions the performance of social distancing, is the ghostly apparition of man’s ends, divested of any instrumental teleology, as pure absence (i.e. death). It is in proximity to the space in-between that social distancing is performed. By dint of prioritizing the proxemic mode over the linguistic one, the meaning of this univocally branded interim space as branded place may not be captured by either subjectivist or objectivist accounts, but through experiential marketing categories. that seek to encapsulate immersive experiences that pass under the radar of the res cogitans.
